# Internal Microstructure Dictates Interactions of Polymer-grafted Nanoparticles in Solution

**DOI:** 10.1021/acs.macromol.1c00907

**Published:** 2021-07-28

**Authors:** Leo Gury, Samruddhi Kamble, Daniele Parisi, Jianan Zhang, Jaejun Lee, Ayesha Abdullah, Krzysztof Matyjaszewski, Michael R. Bockstaller, Dimitris Vlassopoulos, George Fytas

**Affiliations:** † Institute of Electronic Structure and Laser, FORTH, University of Crete, 70013 Heraklion, Greece; ‡ Department of Materials Science and Technology, University of Crete, 70013 Heraklion, Greece; § Department of Materials Science and Engineering, Carnegie Mellon University, 5000 Forbes Avenue, Pittsburgh, Pennsylvania 15213, United States; ∥ Chemistry Department, Carnegie Mellon University, 4400 Fifth Avenue, Pittsburgh, Pennsylvania 15213, United States; ⊥ Max Planck Institute for Polymer Research, Ackermannweg 10, 55128 Mainz, Germany

## Abstract

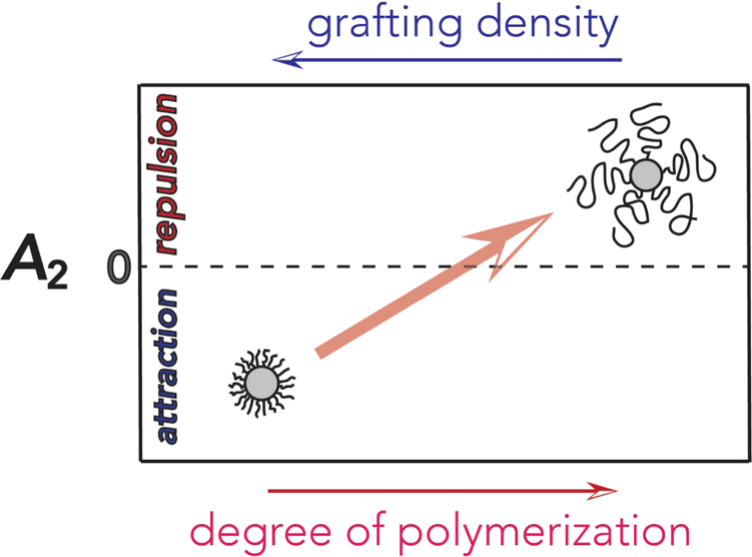

Understanding the effects of polymer brush architecture on particle interactions in solution is requisite to enable the development of functional materials based on self-assembled polymer-grafted nanoparticles (GNPs). Static and dynamic light scattering of polystyrene-grafted silica particle solutions in toluene reveals that the pair interaction potential, inferred from the second virial coefficient, *A*_2_, is strongly affected by the grafting density, σ, and degree of polymerization, *N*, of tethered chains. In the limit of intermediate σ (∼0.3 to 0.6 nm^–2^) and high *N*, *A*_2_ is positive and increases with *N*. This confirms the good solvent conditions and can be qualitatively rationalized on the basis of a pair interaction potential derived for grafted (brush) particles. In contrast, for high σ > 0.6 nm^–2^ and low *N*, *A*_2_ displays an unexpected reversal to negative values, thus indicating poor solvent conditions. These findings are rationalized by means of a simple analysis based on a coarse-grained brush potential, which balances the attractive core–core interactions and the excluded volume interactions imparted by the polymer grafts. The results suggest that the steric crowding of polymer ligands in dense GNP systems may fundamentally alter the interactions between brush particles in solution and highlight the crucial role of architecture (internal microstructure) on the behavior of hybrid materials. The effect of grafting density also illustrates the opportunity to tailor the physical properties of hybrid materials by altering geometry (or architecture) rather than a variation of the chemical composition.

## Introduction

Grafted nanoparticles (GNPs) are made of a solid inorganic core covered by polymer chains tethered to the core surface. GNPs have been obtained using silica,^1−3^ titania,^4^ or plasmonic metallic^5,6^ core materials with a spherical or anisotropic shape,^7^ such as ellipsoidal, rod-like, flat, or even more complex.^8,9^ The submicron size of the grafted particles renders them susceptible to Brownian motion so that they can form stable suspensions in a medium. Due to their small size, GNPs are also sensitive to microscopic forces such as electrostatic, depletion, or Van der Waals. The interplay between the attractive forces and thermal noise governs the stability of colloidal suspensions. If attractions are too strong, the particles aggregate, and at a certain concentration range (depending on the range of attraction), they can form gels.^10,11^ If the interactions between the particles are repulsive, the suspensions are stable. With increasing concentration, the particles can form colloidal crystals^11−14^ and (metastable) glasses,^11,12,15−18^ or liquid crystalline phases^19−21^ if they are anisotropic. Here, we focus on the interactions in the dilute regime, which are of course relevant to the behavior of concentrated suspensions.

Since GNPs are inherently hybrid materials, their properties in solution depend on the mutual contributions of the core and the grafted chains. For GNPs with high grafting density, the dispersion characteristics are usually determined by considering the characteristics of the polymeric shell (graft). While various core materials can be used, the most common is silica. The polymer layer constituting the shell of GNPs is selected based on the desired properties and applications. Current synthesis methodologies offer a broad range of approaches for grafting hydrophilic (poly(ethylene oxide),^22,23^ poly(propylene oxide),^24,25^ poly(methyl methacrylate)^26−28^), hydrophobic (polystyrene,^29−31^ polyisoprene,^32,33^ polybutadiene^34,35^), or responsive (often called “smart”) polymers such as poly-*N*-isopropylacrylamide^36^ (PNIPAM) or poly(2-(dimethylamino)ethyl methacrylate)^37,38^ (PDMAEMA), which can change their interactions with the suspending medium in response to an external stimulus such as pH or temperature.^39,40^ The grafting of such polymers allows for the design of functional materials, the properties of which can be modulated by variation of the suspending medium.

The potential ability to tailor the interactions and properties of hybrid materials can have a substantial impact not only on technology (for example, for improving gas permeability membranes^41,42^ or processing nanocomposites^43^) but also, and more importantly, on the emerging field of biopharmacy, which involves polymer-modified natural materials (such as proteins) with a specific function. Understanding the link between structure solubility in these products is crucial; hence, work with simpler, well-characterized materials can provide insights, as done recently for polymer-tethered enzymes.^44^ It is thus important to explore and exploit the properties of model, well-characterized experimental systems. To this end, investigations with GNPs under various conditions have been at the forefront of soft matter research. In particular, recent work has focused on elucidating the effect of polymer grafting on the interactions between particles in solutions and the solid state.^45−47^ Light scattering studies in solution have suggested that the presence of polymer grafts introduces softness into the particles compared to hard spheres; however, a detailed understanding of the effects of polymer modification on the solution properties (and interactions) of GNPs is lacking. The understanding of the effect of architecture on the interactions between soft GNPs in solution is therefore essential for further developing GNPs into a platform for functional material design. It is exactly this challenge that we address in this work. Assuming fixed core and solvent characteristics, the interactions between GNPs can be tuned by variation of the grafting density, σ, and the chain degree of polymerization, *N*.^48−52^ To facilitate control of these parameters, the material model system in this study is thus comprised of GNPs with the same silica (SiO_2_) core and polystyrene (PS) grafts. The core radius is constant *R*_c_ = 57 ± 4 nm, and the grafting densities range from 0.08 to 0.61 chains/nm^2^ while *N* is in the range 130–2700. Specifically, GNPs of low grafting density (0.1 < σ < 0.3 nm^–2^) with longer chains are expected to exhibit softer behavior characterized by a broader-ranged pair correlation function and pronounced liquid-like ordering compared to hard-sphere suspensions, and this is true both for GNP suspensions and GNP melts (self-suspended).^53−59^ To determine the effect of graft composition (number and size of grafted chains) on the interactions between particles in solvents, we utilize combined static and dynamic light scattering in conjunction with full form factor analysis to determine the second virial coefficient (*A*_2_) and the translational diffusion coefficient of model GNPs in an athermal solvent for PS. From *A*_2_, the pair interaction potential is determined and compared to a brush potential derived for grafted spheres in solution. The results highlight geometry as an equally important parameter in determining the properties of GNPs in solution as compared to chemical composition, and thus suggest new opportunities for controlling the properties of hairy particles. At a more fundamental level, they can serve to exploit this new path to affect thermodynamic properties in chemically identical materials through a change of the internal microstructure (monomer density distribution, segmental conformation).

## Experimental Section

### Materials

Styrene (S, 99%, Aldrich) was purified by passing through a column filled with basic alumina to remove the inhibitor. Tris(2-dimethylaminoethyl)amine (Me6TREN, 99%, Alfa), 4,4′-dinonyl-2,2′-bipyridyne (dNbpy, 97%, Aldrich), anisole (99%, Aldrich), tetrahydrofuran (THF, 99%, VWR), methanol (99%, VWR), hexane (99%,VWR), acetone (99%, VWR), N,N-dimethylformamide (DMF, 99%, VWR), 2-bromoisobutyryl bromide (2BiB, Aldrich, 98%), triethylamine (TEA, Aldrich, 99.5%), copper(II) bromide (CuBr2, 99%, Aldrich), copper(II) chloride (CuCl2, 99%, Aldrich), copper(I) chloride (CuCl, 97%, Sigma-Aldrich), tin(II) 2-ethylhexanoate (Sn(EH)_2_, 95%, Aldrich), hexane (Fluka), 48% hydrofluoric acid aqueous solution (HF, >99.99%, Aldrich), ammonium hydroxide aqueous solution (NH_4_OH, 28.0–30.0%, Fisher), anhydrous magnesium sulfate (MgSO_4_, Fisher), and hexamethyldisilazane (HMDZ, Aldrich, 99%) were used as received unless otherwise stated. Copper(I) bromide (CuBr, 98%, Acros) was washed with glacial acetic acid to remove any soluble oxidized species, filtered, washed twice with anhydrous ethyl ether, dried, and kept in a vacuum. Silica (SiO_2_, with an effective radius *R*_c_ = 57 ± 4 nm as measured by TEM) 20 wt % colloidal dispersion in methyl isobutyl ketone (MIBK-ST) was donated by Nissan Chemical America Corp. The initiator 3-(chlorodimethylsilyl)-propyl 2-Bromoisobutyrate (BiBSiCl). BiNSiCl was synthesized by the reaction of allyl 2-bromoisobutyrate with chlorodimethylsilane, as described elsewhere.^60^ After the extraction of the catalyst and removal of unreacted silane by distillation, the product was obtained as a yellow liquid. ^1^H NMR (300 MHz, CDCl3) spectrum δ: 4.18 (t, *J* = 6.7 Hz, 2H), 1.94 (s, 6H), 1.86–1.78 (m, 2H), 0.93–0.83 (m, 2H), 0.44 (s, 6H) ppm. Surface Modification of Silica NPs. Coupling of the initiator to the surface of silica nanoparticles was achieved by slow injection of 1.5 mL of initiator into 10 mL of silica particle dispersion under stirring. After 24 h of stirring (60 °C) and cooling down to room temperature, 1.1 mL (5.4 mmol) of HMDZ was slowly injected, followed by stirring for 12 h (35 °C). The pale brown dispersion was dialyzed against methanol (3×) and acetone (2×) prior to further use. SI-ATRP of surface-modified nanoparticles was performed following the previously published procedures.^61^

Number-average molar masses and molar mass distributions were determined by size-exclusion chromatography (SEC). The SEC was conducted with a Waters 515 pump and Waters 410 differential refractometer using PSS columns (Styrogel 105, 103, 102 Å) in THF as an eluent at 35 °C and at a flow rate of 1 mL min^–1^. Linear PS standards were used for calibration. Thermogravimetric Analysis (TGA) was performed to measure the fraction of SiO_2_ in the hybrids (TA Instruments 2950). The heating procedure involved four steps: (1) jump to 120 °C; (2) hold at 120 °C for 10 min; (3) ramp up at a rate of 20 °C/min to 800 °C; and (4) hold for 2 min. The use of surface-initiated ATRP as the synthesis method^1,30,33,40,55^ allows for the dense grafting of long polymer chains on the surface. The various molecular characteristics of the utilized polystyrene-grafted silica nanoparticles (abbreviated as SiO_2_@PS) are listed in Table 1.

**Table 1:** Molecular Characteristics (Degree of Polymerization N, Grafting Density σ, PS Arm Weight-Average Molar Mass *M*_a_, and Total Weight-Average Molar Mass of the GNP Chains, *M*_w_), Structural Parameters (Hydrodynamic *R*_h_ and Particle *R*_m_ Radii of the Polystyrene (PS)-Grafted Silica Nanoparticles, and Shell Volume Fraction ϕ_shell_), and Interaction Parameter (*A*_2,exp_) in Dilute Dispersions in Toluene at 293 K

code	*N*	*M*_a_ (kg mol^–1^)	*M*_w_ × 10^–6^^a^ (kg mol^–1^)	σ (nm^–2^)	*R*_h_ (nm)	*R*_m_ (nm)	ϕ_shell_^b^	*A*_2,exp_ (mol cm^3^ g^–2^)
DP130	135	14	1.3	0.61	97	80	0.261	–5.6 × 10^–7^
DP440	442	46	2.1	0.61	123	125	0.223	–2.7 × 10^–6^
DP790	788	82	2.7	0.52	170	155	0.178	–5.0 × 10^–7^
DP980	980	102	3.0	0.49	224	171	0.156	–2.4 × 10^–6^
DP2690	2692	280	6.3	0.47	436	343	0.051	2.5 × 10^–5^
DP480	480	50	1.8	0.3	140	180	0.053	–1.3 × 10^–9^
DP1170	1170	122	1.6	0.08	145	146	0.065	8.4 × 10^–9^
DP1300	1300	135	4.3	0.53	190	171	0.297	1.8 × 10^–8^
DP2480	2480	258	5.5	0.39	430	227	0.178	1.5 × 10^–7^

a Based on TGA measurements.

b ϕ_shell_ = *V*_shell_/*V*_GNP_ = 3σ*M*_w_*R*_c_^2^/(ρ_PS_*N*_A_*R*_m_^3^).

### Light Scattering

We used a commercial ALV-5000 (Germany) instrument equipped with a goniometer. The scattering angle, θ, between the incident and the scattered light, varying between 18 and 150°, determines the magnitude of the scattering wave vector , where *n*_0_ is the refractive index of the solvent. A Nd-YAG laser with a wavelength of λ = 532 nm (green) and a power of 120 mW was used as the light source. Filters were added on the optical path as needed to lower the incident power for strongly scattering samples. The temperature of the bath containing the sample was fixed at 293 K for all experiments. Static polarized light scattering was used to characterize the size and the interactions of dilute solutions of grafted nanoparticles.^62^ The average intensity *I*(*q*,*c*) of the light scattered from the grafted nanoparticle solution in toluene was measured as a function of the scattering wave vector *q*, and concentration, *c*, and transformed to the absolute Rayleigh ratio *R*_VV_ (*q*,*c*) = [*I*(*q*,*c*)/*I*_T_] *R*_T_, with *I*_T_ and *R*_T_ (=2.78 × 10^–5^ cm^–1^ at 532 nm and 20 °C) being, respectively, the intensity and Rayleigh ratio of the solvent toluene. In the limit *q* → 0
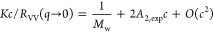
1where  is a numerical factor depending on the light and solution properties, *M*_w_ is the weight-average molar mass of the scattering object, and *A*_2,exp_ is the second coefficient of the virial expansion. The other physical quantities are the Avogadro number *N*_A_ and the refractive index contrast d*n*/d*c*, computed from the absolute *R*_VV_ (*q* →0)/c and the GNP *M*_w_ (core plus grafted PS).^62^ The optical contrast drops from 0.11 Lkg^–1^ in PS/toluene to 0.043 Lkg^−1^ in DP130/toluene with the highest SiO_2_ fraction (see also Figure S1 of the Supporting Information); note that the value of *A*_2_ is independent of the value of d*n*/d*c*, which affects only the absolute Rayleigh ratio.

For the dilute GNP solutions, the *I*(*q*) pattern was described by the core and inhomogeneous shell model, representing the hard silica core and the solvent-swollen polystyrene shell of the particles, respectively. It was implemented in “Scatter” software,^63^ which was used to fit the data with the main input being the radius of the silica core (details are shown in the Supporting Information, S2). A typical *I*(*q*) profile for 0.0332 wt % DP1170 in dilute toluene solution at 293 K is shown in Figure S2. The form factor of a core–shell spherical object was taken as^60^*P*(*q*) = *F*^*2*^(*q*), where
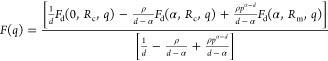
2where *R*_c_ is the radius of the core, *R*_m_ is the radius of the grafted nanoparticle, *d* = 3, *p* = *R*_c_*/R*_m_, and α denotes a free parameter (it takes a value of zero for homogeneous shells of constant density). For the core (hard sphere), we have  and the rest of the parameters are , with *n* being the refractive index in the different regions, ϕ_c_ = (*R*_c_/*R*_m_)^3^, and ϕ_cs_ = *V*_shell_/*V*_GNP_ = 3σ*M*_w_*R*_c_^2^/(ρ_PS_*N*_A_*R*_m_^3^).

Dynamic light scattering (DLS) was performed on dilute samples of the SiO_2_@PS samples. DLS records the autocorrelation of the time-dependent scattered intensity, , where the brackets denote a time average value and the decay time spans the range 10^–7^ < *t*(s) < 10^3^.^62^ In the simple case of dilute solutions of Brownian particles (at rest), the decay of the field autocorrelation function, *C*(*q*,*t*) = [*G*(*q*,*t*) – 1]^1/2^ = *b* exp[−*t*/τ(*q*)], is well captured by a single exponential decay (Figure S3). The amplitude *b* ≤ 1 is the fraction of total scattering intensity relaxing at *t* > 10^–7^s. The relaxation time is determined by the diffusion coefficient  of the GNPs. The overlap concentration, c*, defined as the solution concentration beyond which grafted particles start to touch each other, is approximately given by . All measurements in this study were performed at concentrations in the range between 3 × 10^–4^c* and 4 × 10^–2^c*, well within the dilute regime; hence, the *I*(*q*) patterns measure the form factor of the individual GNPs.

## Results and Discussion

### Form Factor and Dimensions

**Figure 1:**
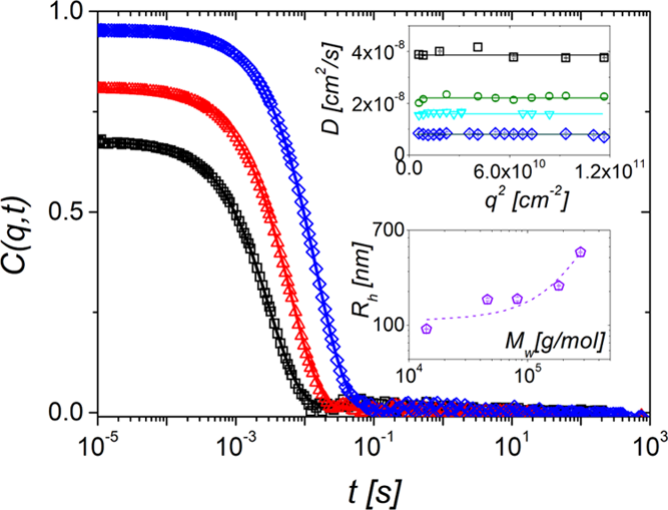
Intermediate scattering function C(q,t) of three SiO_2_@PS GNP dilute solutions in toluene at a scattering wave vector *q* = 9.14 × 10^–3^ nm^–1^ (scattering angle 30°): DP130 (*c* = 2.45 × 10^–4^ g/cm^3^) open squares; DP790 (1.48 × 10^–5^ g/cm^3^) open triangles; DP2690 (1.28 × 10^–4^ g/cm^3^) open diamonds. The solid lines represent the fits of the experimental *C*(*q*,*t*) by a single exponential decay function. Upper inset: extracted translation diffusion coefficient *D* plotted against *q*^2^, where the lines denote the average *D* for DP130 black open squares, DP440 open green circles, DP980 open light cyan down-triangles, and DP2690 open blue diamonds. Lower inset**:** the hydrodynamic radius, *R*_h_ as a function of the total weight-average molar mass *M*_w_ of the GNP chains. The radius of the SiO_2_ core is the same (57 ± 4 nm) for all GNPs.

The hydrodynamic radius, *R*_h_, of each sample was obtained from the relaxation function *C*(*q*,*t*) at low *q* in the dilute regime. Figure 1 displays the experimental *C*(*q*,*t*) for three SiO_2_@PS GNPs in toluene at a low scattering wave vector. The increase of the amplitude *b* of *C*(*q*,*t*) from DP130 to DP2300 indicates the increasing contribution of the scattering from the particle brush to the total light scattering (including the solvent contribution) due to its growing size in the same direction. The relaxation functions *C*(*q*,*t*) are well represented (solid lines) by a single exponential decay function with rate Γ(*q*) = *Dq*^2^. The translation diffusion coefficient (*D* = *D*(*q*)) is virtually independent of *q* (upper inset to Figure 1) and yields the hydrodynamic radius via the Stokes–Einstein–Sutherland equation, *R*_h_ = *k*_B_*T*/(6πη*D*), where *k*_B_ and η are, respectively, the Boltzmann’s constant and the solvent (toluene) viscosity. The total size of the particles represented by *R*_h_ expectedly increases with the particle *M*_w_ as shown in the low inset to Figure 1. Since the radius of the SiO_2_ core is the same for all SiO_2_@PS particles, *R*_h_ depends on the grafting density, the chain length, and the solvent quality. This dependence will be discussed in Figure 3 below.

**Figure 2:**
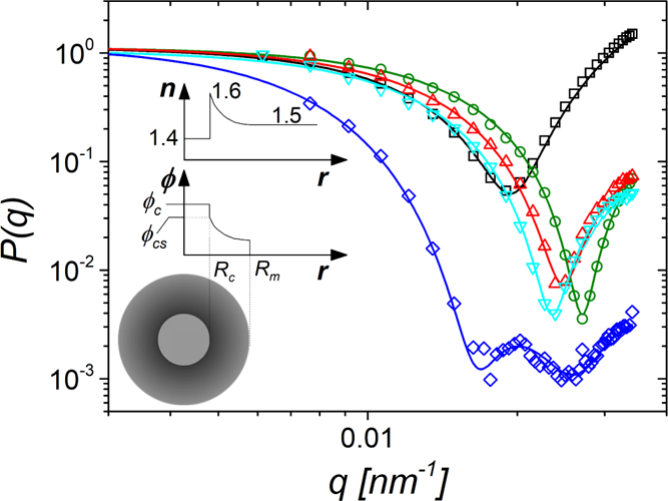
The form factor *P*(*q*) obtained from the static light scattering is shown for three SiO_2_@PS GNP dilute solutions in toluene: DP130 (*c* = 2.45 × 10^–4^ g/cm^3^) gray squares, DP440 (1.48 × 10^–5^ g/cm^3^) green circles, DP790 (1.48 × 10^–5^ g/cm^3^) red up-triangles, DP980 (1.1 × 10^–4^ g/cm^3^) cyan down-triangles, DP2690 (1.28 × 10^–4^ g/cm^3^) blue diamonds. The lines through the data are the best fits to the data by means of “Scatter” software^63^ (see the text). Inset: illustration of the refractive index (top) and density (middle) profiles along with an illustration of the grafted sphere with the respective gradients throughout its volume (bottom).

In contrast to the robust and *q*-independent *D*, the light scattering intensity *I*(*q*) depends on *q*^–1^ as already expected by the sizable *R*_h_ and the increase of the amplitude, *b*, of *C*(*q*,*t*) in Figure 1. Hence, submicron SiO_2_@PS particles allow access to their form factor, *P*(*q*) (= *R*_VV_(*q*)/*R*_VV_(*q* = 0)) from the *q*-dependent polarized *R*_VV_(*q*), as shown for four systems in Figure 2. Light scattering allows capturing the *P*(*q*) of the present GNPs even beyond the first interference minimum (e.g., for the largest GNP in this figure). Hence, a reliable estimation of the density profile is possible, as discussed below. The second peak is the higher-order interference peak. The *R*_VV_(*q*) data were fitted by means of “Scatter” software.^63^ The refractive index *n*(*r*) and the monomer density profile φ_*cs*_(*r*) were determined by the software and are shown as upper and lower insets to Figure 2 together with an illustration of the core–shell particle topology. The size dispersity of the core was an adjustable parameter and was found to vary between 13% (DP130) and 5% (DP2690) in accordance with electron imaging results (not shown here). Note that the appearance of a clear single or even double (for DP2690) minimum indicates a rather uniform particle size. The contrast parameter and the GNP size *R*_m_ are additional adjustable parameters in the representation of the experimental *P*(*q*) by eq 2. The monomer density φ(*r*) in the inhomogeneous PS shell was modeled as a power law with a fixed value of the exponent α in eq 2. In the case of spherical brushes, the theoretical prediction is α = −4/3.^52,64,65^ Only for DP2690, the density profile was steeper and represented by α = −1.57. As depicted in Figure 2, for the brush system with the largest size *R*_m_ (DP2300), the second-order peak was also resolved within the light scattering *q*’s. Notably, its location is ∼3^1/2^ higher than the first minimum as for the case of hard spheres. The size, *R*_m_, for all GNP’s is listed in Table 1.

**Figure 3:**
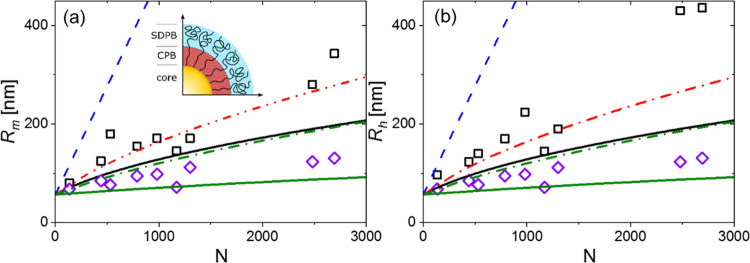
(a) Radius *R*_m_ obtained from the form factor of the grafted SiO_2_@PS as a function of the PS degree of polymerization *N*. Black squares represent the SiO_2_@PS GNPs. The violet open diamonds are the calculated values of the collapsed size based on the density of PS (1060 kgm^–3^). The blue dashed line represents the size at the maximum extension of the grafted chains (using the effective monomer size of *l* = 0.44 nm). The continuous black and green lines are the model prediction of Ohno et al.^49^ at the CPB limit for a grafting density of 0.6 and 0.08 nm^–2^, respectively (eq 3), again with *l* = 0.44 nm. The red and green dashed-dotted lines correspond to the predictions at the SDPB limit for a grafting density of 0.6 and 0.08 nm^–2^, respectively, calculated using eq 4 (the ratio *R*_m_/*R*_h_ is plotted as a function of *N* in Figure S4). Inset: illustration of a section of a GNP with the three regimes, core, CPB, SDPB (see the text). (b) Respective plot of a hydrodynamic radius as a function of the degree of polymerization. Definitions of symbols and lines are the same as in (a).

To appreciate the internal microstructure revealed by the data in Figure 2, it is instructive to review the pertinent information from the literature. Ohno et al.^49^ described a model with two regimes that depend on the monomer density in the brush, which derives from the Daoud and Cotton model^64^ and is appropriately adapted to estimate the size of polymer-grafted particles. It assumes that the conformation of the grafted chains differs based on the proximity of their neighbors and, therefore, depends on the grafting density, chain length, and solvent quality. The chains can therefore be in the concentrated polymer brush (CPB) regime, in which the excluded volume interactions are screened by the stretching of the chain. This occurs when the grafting density is very large, and the chains are short, and/or the solvent is bad. The transition toward the semidilute polymer brush (SDPB) regime occurs when the grafted chain is sufficiently long to experience excluded volume interactions. The model provides a theoretical cut-off length beyond which the chains should cross over from the CPB into the SDPB regime. Alternatively, it is possible to estimate the size of the particles based on molecular parameters, assuming one or the other regime, which is what is illustrated in Figure 3. Based on scaling arguments,^49^ the cut-off size between CPB for *r* < *R*_cross_ and SDPB for *r* > *R*_cross_ was derived as *R*_cross_ = *R*_*c*_σ_0_^1/2^υ_0_^–1^, where σ_0_ is the normalized grafting density σ_0_ = σ*S*, *S* is the monomer surface area, and υ_0_ is the normalized excluded volume parameter. It depends on the solvent quality (, with υ = 1/2 – χ, where χ ≈ 0.37 for PS/toluene^66^) and *S* = 0.62 nm^2^ is the cross-sectional area of the PS unit (from which an effective monomeric size of  = 0.44 nm is estimated^49,67^).

In Figure 3, different cut-off values and fractions of overall GNP size are used, and they are listed in Table S1. The GNP size represented by *R*_m_ and *R*_h_ (Table 1) is plotted as a function of the degree of polymerization *N* of the grafted PS chain and compared with the predictions of the CPB (solid black line, eq 3) and SDPB (dashed-dotted line, eq 4) regimes.^49^ To appreciate these real dimensions, the sizes for two extreme graft conformations, stretched (dashed blue line) and collapsed chains (with ρ_PS_ = 1060 kg/m^3^, violet diamonds), are also shown in Figure 3.
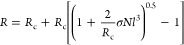
3
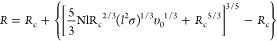
4It appears that, overall, the present GNPs conform to the SDPB regime, but we note that the estimated fraction of the CPB regime (which we may call the dry regime) is much more significant for DP130 (see Table S1). We also point out that recently the brush conformation of GNP melts (ideal case) was investigated by simulations and modeling,^68^ expanding original ideas developed for star polymers,^69,70^ and a qualitatively similar behavior was revealed, i.e., two regimes inner dry and outer wet.

The size measured with static light scattering is somewhat different from the hydrodynamic radius (which is an apparent size) obtained by dynamic light scattering, as expected for this type of particles. We used the same analysis described above based on the Ohno et al. model^49^ for grafted nanoparticles (Figure 3). The rest of the data are in very good agreement with the theoretical model, with a progressive transition from the CPB at a low degree of polymerization to the SDPB at higher degrees of polymerization. Concerning the clear discrepancies at large values of *N*, it should be noted that the above model was developed for static size, while the hydrodynamic size is apparent and the solvent effect is significant at large *N*. In addition, the crossover between dry and wet regimes may be broader than considered in the model and the exact transition cannot be determined accurately at present without additional evidence (for example, by neutron scattering and simulations). Moreover, the grafting density of the experimental GNPs is not constant. The punchline here is that the present GNPs appear to conform to the SDPB regime.

### Second Virial Coefficient

**Figure 4:**
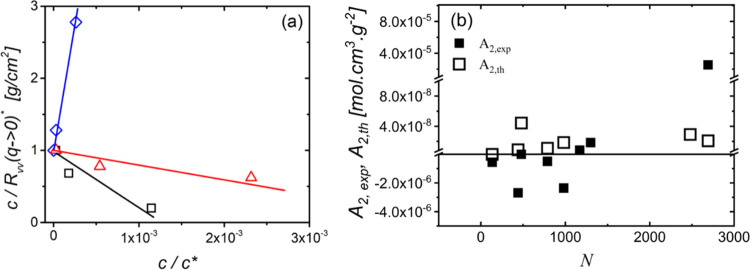
(a) Concentration dependence of the reciprocal scattering intensity in the limit of q->0 for SiO_2_@PS GNPs with *N* = 130 (squares), *N* = 788 (triangles), and *N* = 2300 (diamonds). (b) Second virial coefficient for the different GNPs of Table 1 as a function of *N*, extracted from experiments (solid squares) and theoretical calculations (eq 6) (see the text and Table S2).

The previous section discussed the form factor P(q) and provided a thorough characterization of both the internal microstructure and overall size, which supports the quality of the experimental GNP samples. This provided the needed ingredients for exploring the nanoparticle interactions, which we do now. The analysis of the evolution of the scattered intensity with the concentration provides information about the interactions in the dilute regime via the determination of the second virial coefficient *A*_2,exp_. In the limit of the low scattering wave vector, *q*, and dilute conditions, *A*_2,exp_ can be determined from the concentration dependence of *Kc*/*R*_VV_(*q* → 0). Figure 4a shows the variation of *c*/*R*_VV_(*q* → 0) with concentration for SiO_2_@PS GNPs dilute dispersions in toluene with three PS grafts (*N* = 130,788 and 2300). The linear representation of *c*/*R*_VV_(*q* → 0) vs *c* allows the estimation of *A*_2,exp_ (eq 1). The slope of this plot provides important qualitative information about the nature of the total interactions between particles in the dilute regime. The experimental values found for the GNPs are reported in Table 1.

**Figure 5:**
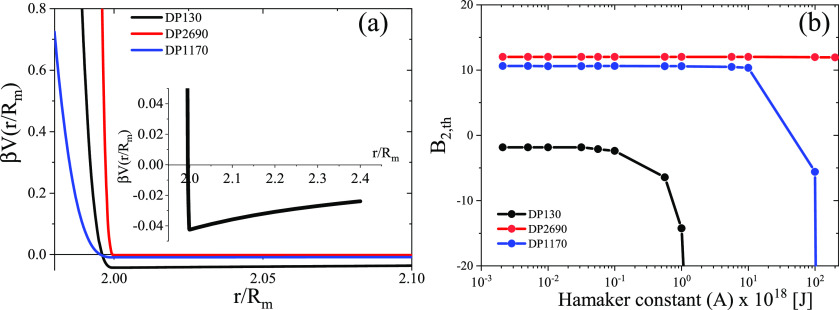
(a) Calculated interaction potentials for the SiO_2_@PS particles, DP130 (black line), DP1170 (blue line), and DP2690 (red line). The vertical axis shows the strength of the interactions and is multiplied by β = 1/*k*_B_*T*. The horizontal axis represents the center-to-center distance between two particles divided by their radius *R*_m_. The inset represents a close-up of the potential for the DP130 sample. (b) Parametric analysis of the calculated dimensionless second virial coefficient *B*_2_,_th_ for DP130 (black), DP1170 (blue), and DP2690 (red).

In Figure 4b, we assess the experimental information in view of the theoretical expectations for the interactions. The plot separates positive from negative values of the second virial coefficient through the horizontal dividing line. There are four experimental samples with negative A_2_ (solid squares), whereas the sign of *A*_2,exp_ is uniquely defined (increase or decrease of *c*/*R*_VV_, see Figure 4a), its small value is subject to a relatively large error (it can easily reach 50%). The increase of the positive *A*_2,exp_ for GNPs with large PS composition (increasing *N*) toward the PS value is expected. The theoretical *A*_2,th_ values (open squares) are predicted to be positive for all GNPs but DP130, based on the assumed potential (see Figure 5b and discussion below). Based on this, it seems unexpected for PS in good solvent conditions that *A*_2,exp_ is found to be small and negative for the samples with high grafting density and low degrees of polymerization compared to much larger and positive *A*_2,exp_ values (∼4 × 10^–4^ mol cm^3^g^–2^) of pure PS in the same solvent (Figure 4b). This suggests an overall attractive interaction between these particles. However, this attraction is weak and does not lead to aggregation or phase separation in the solutions at these concentrations and under the given experimental conditions as confirmed by the single-particle diffusion coefficient extracted from the exponential *C*(*q*,*t*) functions (Figure 1). In the case of the GNPs with higher degrees of polymerization, *A*_2,exp_ becomes positive, meaning that the net interaction turns weakly repulsive. It is the dependence of the sign of the second virial coefficient on grafting density and, in particular, its unambiguous change of sign that represents the main finding of this work.

To interpret the results, the second virial coefficient was computed from the pair interaction potential. To this end, we used a brush model, which we have appropriately modified to account for attractive interactions due to Van der Waals forces. The model was originally developed by Likos and co-workers to describe interactions between colloidal particles with adsorbed stabilizing layers^70^ and was recently assessed systematically for GNPs by some of us.^52^ The modification described below was inspired by the analysis of interacting star polymers in solvents of varying quality.^50^ Hence, the model comprises the steric repulsion of grafted chains and Van der Waals attraction due to the silica core–core dipole interactions. Finally, an additional hard-core repulsive contribution is added with an energy value high enough to be considered infinite for all calculations. The full expression for *V*(*r*) normalized by β = 1/*k*_B_*T* reads
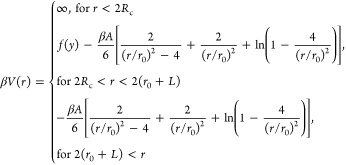
5where 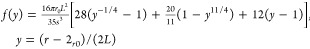
*L*(=*R_h_* − *R_c_*) is the thickness of the brush layer, *s* = σ^–1/2^, *A* is the Hamaker constant 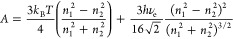
and *n*_1_ = 1.6 and *n*_2_ = 1.4 are the refractive indices of silica and the PS brush layer, respectively (note that for toluene *n* = 1.5), *h* = 6.63 × 10^–34^ Js is the Planck constant, and *ν*_e_ is the frequency of the incident light (*ν*_e_ = 5.64 × 10^14^ s^–1^ at 532 nm). These values yield an average Hamaker constant *A* = 2.27 ×10^–21^ J. Using the potential of eq 5, we can now calculate a dimensionless theoretical value of the second virial coefficient, *B*_2,th_.^50^
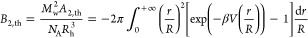
6The integral over the center-to-center distance *r* is approximated by a sum over all the distance increments d*r* = 0.1 nm. We used *R* = *R*_m_ from Table 1, and the computed *A*_2,th_ values are reported in Table S2.

Characteristic theoretical interaction potentials for some of the GNPs studied here are depicted in Figure 5a (see also Figure S5a). We observe that the potential of the particles with the smallest degree of polymerization DP130 (black line in Figure 5a) exhibits a weak, albeit unambiguous, short-range attraction, in contrast to the rest of the particles. On the other hand, due to its substantially lower grafting density compared to the other samples, DP1170 exhibits a repulsive potential with a much broader increase (Figure 5a), indicative of a softer particle. The rest of the samples (Figure S5a) seem to be very similar in terms of the interaction potential. To appreciate the link between the internal particle microstructure (as described by σ and *N*) and the interaction potential, we performed a simple parametric study where we varied the effective attractions by changing the value of the Hamaker constant *A* somehow arbitrarily from 10^–21^ to 10^–16^ J. We can see from Figure 5b that the attractions between brush particles with a realistic value of *A* are non-negligible, albeit weak, only in the case of DP130. In fact, a significant impact of core–core interactions on *B*_2,th_ is expected only if *A* would be to change by more than 2 orders of magnitude. Along these lines, the model shows that DP1170 nanoparticles would need to experience an even larger *A* (exceeding the set value by 4 decades) to result in attraction. The same analysis for the other GNPs is presented in Figure S5b. Note that the value of the Hamaker constant *A* impacts the sign of *A*_2_ only for the sparsely grafted nanoparticles (DP1170 in Figure 5b) with patchy core surfaces,^57^ whereas the sign of the second virial coefficient is robust to the variation of *A* in the case of densely grafted nanoparticles (DP2690, DP130). Therefore, the negative second virial coefficient is not the consequence of bare core–core attraction, as determined by the value of *A*, but it is the grafted layer-mediated microstructure with predominantly stretched chain conformation (fraction of the dry region^68^) that plays the key role (e.g., in DP130).

Although this simple model does predict a negative value of *A*_2,th_ for GNP DP130 with the lowest *N* and highest σ, it serves here only as a qualitative confirmation of the intriguing experimental findings. Several details such as size dispersity, polar interactions of the surface, or surface inhomogeneity, are not considered (note that solvent–segment interactions are neglected). Also, we only used an ad-hoc expression for the attractive part of the pair interaction potential and considered a Hamaker constant for the SiO_2_@PS system. Furthermore, there is also an uncertainty in the determination of the dry CPB and wet brush SDPB regimes since the calculations in Figure 3 are very sensitive to the choice of monomer size (we used an effective size based on the cross-sectional area, following ref (49)). These limitations could explain the differences between the measured and calculated values of the second virial coefficient for DP2480 (Figure 4b). Nevertheless, the common trend of experimental and calculated results in Figures 4 and 5 illuminates a surprising impact of graft architecture on the solution behavior of tethered particles. Overall, it seems that the core–core attraction and the steric repulsion compensate each other in most of the systems studied here. However, it is clear that an increasing fraction of the dry CPB layer can impart weak attraction and thus a negative second virial coefficient, which is in contrast to considerations based on only graft/solvent composition.

For large values of *N* > 1000, the increase of experimental *A*_2,exp_, accompanied by a weaker increase of theoretical *A*_2,th_, may indicate that the shells become more repulsive. This is consistent with the ratio *R*_cross_*/R*_m_ of Table S1 and can be explained as the shell being less dense and occupying a larger volume with respect to the other samples. Based on the Daoud–Cotton model,^58^ this would result in a larger blob size and stronger excluded volume interactions. Therefore, under the same conditions (solvent, temperature), there is a competition between the attractive Van der Waals forces and the repulsive steric effects, which influences the second virial coefficient. When the shell layer (grafted arms) is short, the Van der Waals forces are not entirely screened by the grafted chains and the second virial coefficient becomes negative (weakly), which is the case for the smallest particle studied here (DP130, Figure S5). Finally, we briefly address the possibility of attractions due to possible heterogeneity in drafting density distribution in the GNPs. Indeed, such a situation results in patchiness, which gives rise to (typically attractive) anisotropic interactions.^71,72,43^ Based on the synthetic procedure and characterization discussed above, this possibility could be safely excluded, especially for higher grafting densities. Concerning the bare silica particles, their entire surface was covered by hydrophobic groups, even for low grafting densities. Moreover, grafting should smear out possible topological heterogeneities of the silica surface. Hence, the internal microstructure of the highly grafted GNPs (and not the patchiness) is at the origin of the thermodynamic change of the solvent quality and resulting change of sign of the second virial coefficient. To this end, it is important to investigate the effects of bulkiness and polarity of the monomers using chemically different grafted polymer chains and/or cores.

## Conclusions

We have shown that the details of the internal microstructure (grafting density, degree of polymerization of grafted chain) significantly affect the interactions of grafted nanoparticles under the same conditions (chemistry, solvent, temperature) in solution. In particular, the measurements of the second virial coefficient reveal that sufficiently dense brush architectures (i.e., in the limit of high grafting density and low degree of polymerization) can trigger attractive interactions even in systems for which dissolution would be expected on the basis of polymer/solvent composition. A simple theoretical analysis based on a coarse-grained brush potential rationalizes the observed trend as a consequence of the balance between attractive core–core interactions and excluded volume interactions imparted by the polymer grafts. The surprising role of brush architecture on interactions in solutions suggests new opportunities for tailoring properties of “brush particle type systems” by designing the microstructure rather than the chemistry of polymer tethers. Of course, we may expect a different dynamic behavior at higher concentrations. However, this represents a challenge to be addressed in the future, and the present dilute solution investigation already reveals the crucial role of the internal microstructure on the properties of grafted nanoparticles.

## Supporting Information Available

Refractive index increment (Figure S1); static and dynamic light scattering data (Figures S2 and S3); sizes of GNPs (Figure S4, Table S1); second virial coefficient (Table S2); calculated interaction potentials (Figure S5) (PDF)
