# Supplementary material for: Internal Microstructure Dictates Interactions of Polymer-grafted Nanoparticles in Solution
Source: Macromolecules. 2021 Jul 28;54(15):7234–43. doi: 10.1021/acs.macromol.1c00907 (PMC8361431; doi:10.1021/acs.macromol.1c00907)
Supplement: Supplementary file 1 — ma1c00907_si_001.pdf [file ma1c00907_si_001.pdf]

SUPPORTING INFORMATION

Internal microstructure dictates interactions of polymer-grafted nanoparticles in solution

Leo Gury[1,2], Samruddhi Kamble[1], Daniele Parisi[1,2], Jianan Zhang[3], Jaejun Lee[3], Ayesha Abdullah[3], Krzysztof Matyjaszewski[4], Michael R. Bockstaller[3], Dimitris Vlassopoulos*[1,2], George Fytas*[5]

[1] Institute of Electronic Structure and Laser, FORTH, 70013 Heraklion, Greece
[2] Department of Materials Science and Technology, University of Crete, 70013 Heraklion, Greece
[3] Department of Materials Science and Engineering, Carnegie Mellon University, 5000 Forbes Ave., Pittsburgh, PA 15213
[4] Chemistry Department, Carnegie Mellon University, 4400 Fifth Ave., Pittsburgh, PA 15213
[5] Max Planck Institute for Polymer Research, Ackermannweg 10, 55128, Mainz, Germany

Correspondence:

G. Fytas (fytas@mpip-mainz.mpg.de), D. Vlassopoulos (dvlasso@iesl.forth.gr)

Contents:

Refractive index increment (Fig.S1); Static and dynamic light scattering data (Fig.S2,S3); Sizes of GNPs (Fig.S4, Table S1); Second virial coefficient (Table S2); Calculated interaction potentials (Fig.S5).

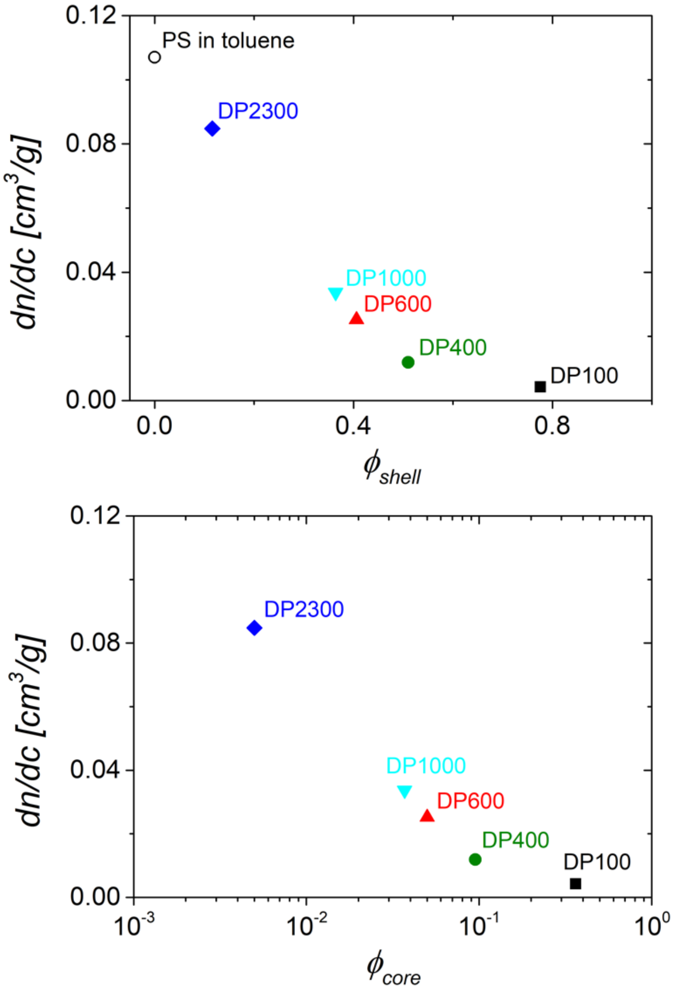

Figure S1. Refractive index increment *dn*/*dc* from the absolute $R_{vv}(q=0)/c$ intensity and the GNP molecular weight M_w (core plus PS brush) vs the PS volume fraction computed from the provided molecular characteristics. Also plot vs ϕ_{core} (0.363 for DP100; 0.095 for DP400; 0.05 for DP600;0.037 for DP 1000; 0.005 for DP2300 (From Table 1).

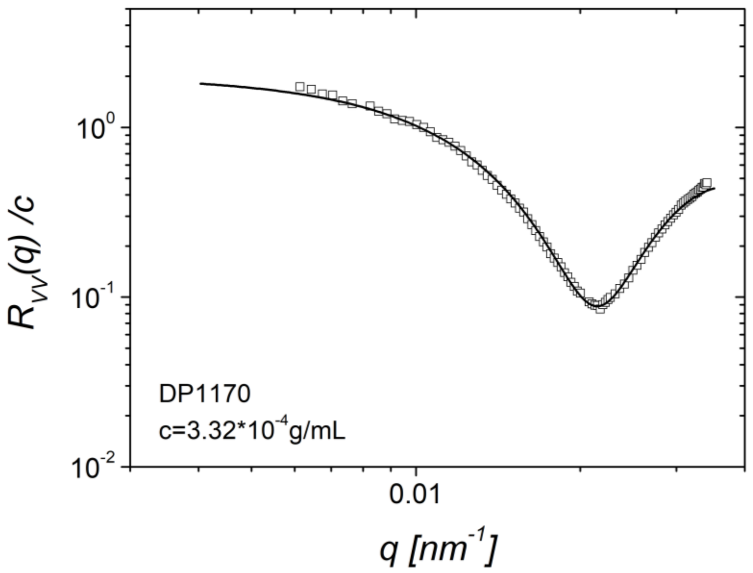

Figure S2. Absolute Rayleigh scattering $R_{VV}(q)/c$ profile for DP1170 sample at $3.32\cdot10^{-4}$ g/mL solution in toluene solution. The solid line denotes the best fit using "scatter" with parameters R_c=59nm (core), σ=0.16, R_m=143nm, α=-1.333, ρ=-0.0582 (optical density of the nanoparticle, see main text).

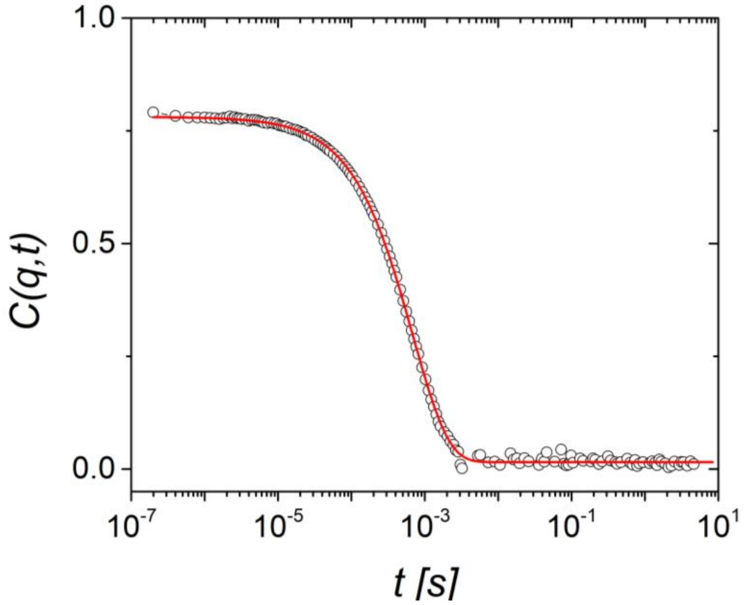

Figure S3. Example of a typical field auto-correlation function of a solution of the DP1300 particle at a scattering angle 90^0 *($q=0.025$ nm^{-1})* and concentration *$1.4\cdot10^{3-4}$g/mL* (highest measured concentration). The red line denotes the representation of the experimental *C(q,t)* by a single-mode exponential decay.

Table S1. Estimated characteristic scales of the grafted nanoparticles: grafting density, normalized grafting density, cut-off length between CBP and SDBP regimes and its ratio to the overall GNP size.

Code	σ (nm^{-2})	σ_0 (nm^{-2})	R_{cross} (nm)	R_{cross} / R_{ross}
DP130	0.61	0.38	38	0.47
DP440	0.61	0.38	38	0.30
DP790	0.52	0.32	35	0.23
DP980	0.49	0.30	34	0.20
DP2690	0.47	0.29	33	0.10
DP480	0.3	0.19	27	0.15
DP1170	0.08	0.05	14	0.10
DP1300	0.53	0.33	35	0.20
DP2480	0.39	0.24	30	0.13

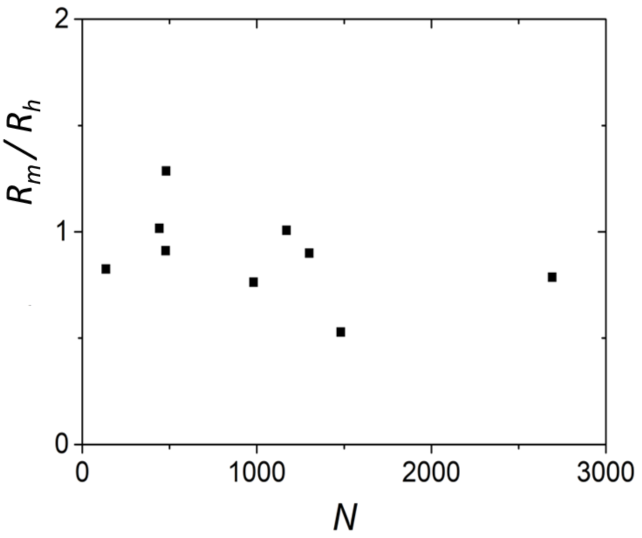

Figure S4. Ratio of R_m/R_h as function of the grafted PS degree of polymerization N.

Table S2. Theoretical second virial coefficient $A_{2,th}$ calculated from eq.6 by using R_m and R_h.

Code	$A_{2,th}$ (R_m) (mol cm^3 g^{-2})	$A_{2,th}$ (R_h) (mol cm^3 g^{-2})
DP130	-4.6x10^{-9}	-8.2 x10^{-9}
DP440	9.4x10^{-9}	8.9 x10^{-9}
DP790	8.3x10^{-9}	1.1 x10^{-8}
DP980	8.2x10^{-9}	1.8 x10^{-8}
DP2690	1.0x10^{-8}	2.1 x10^{-8}
DP480	9.4x10^{-9}	4.4 x10^{-8}
DP1170	1.0x10^{-7}	1 x10^{-7}
DP1300	3.5x10^{-9}	4.8 x10^{-9}
DP2480	4.3x10^{-9}	3.0 x10^{-8}

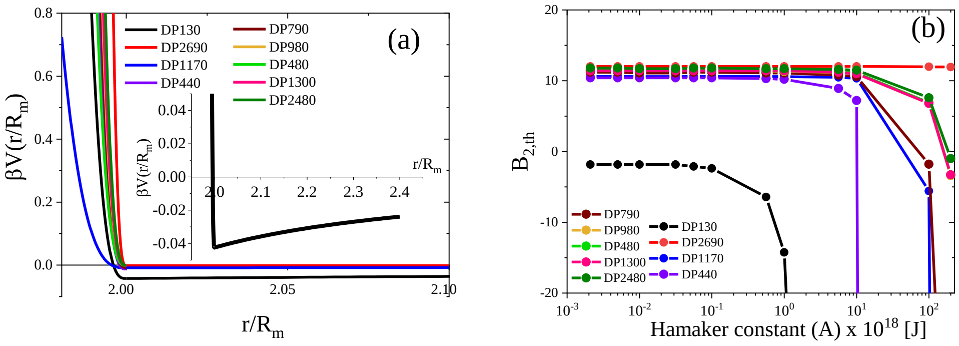

Figure S5. (a) Calculated interaction potential from Eq. 5 for the *SiO₂@PS* particles (see legend). The vertical axis shows the strength of the interactions and is multiplied by $\beta = 1/k_B T$.The horizontal axis represent the center-to-center distance between two particles divided by their radius R_m. The inset represents a close-up of the potential for the DP130 sample. (b) Parametric analysis of the calculated dimensionless second virial coefficient $B_{2,th}$ for the $SiO_2@PS$ particles (see legend).
